# Aspirin resistance among a cohort of Sri Lankan patients

**DOI:** 10.1186/1753-6561-9-S1-A60

**Published:** 2015-01-14

**Authors:** PN Nadarajah, DP Suriyamudalige, BCM Wimalachandra, LV Gooneratne

**Affiliations:** 1 Faculty of Medicine, University of Colombo, Colombo, Sri Lanka; 2 Faculty of Pathology, University of Colombo, Colombo, Sri Lanka

## Background

Aspirin is an effective anti-platelet agent with proven benefit in preventing atherothrombotic complications. However, resistance to aspirin is significantly associated with increased risk of death, cerebrovascular accident or myocardial infarction compared with aspirin sensitive patients (24% vs 10%, P=0.03) and is well documented in Western literature. It has hitherto not been established in Sri Lanka. Our aim was to estimate the prevalence of aspirin resistance in patients on low dose aspirin for primary or secondary prophylaxis and to ascertain if patients resistant to aspirin have detectable serum salicylic acid levels (SA).

## Methods

Platelet aggregometry was performed with Adenosine diphosphate (ADP) and Arachidonic acid in 48 patients on aspirin 150mg daily and 12 normal controls. Serum Salicylic acid levels were also estimated using High Performance Liquid Chromatography (HPLC) on the same blood sample. Aspirin resistance was defined as a mean platelet aggregation of ≥70% with ADP and ≥20% with Arachidonic acid. Aspirin semi responders were defined as those having the above platelet aggregation levels in only one of the two reagents used. Aspirin responders do not show acceptable platelet aggregation with either of the reagents.

## Results

Mean age of patients was 61 years (SD=9.26) with 64% females. 24.4% were aspirin resistant, 64.5% were semi responders and 11.1% were aspirin responders. All semi responders showed normal aggregation with Arachidonic Acid. Salicylic acid levels were successfully performed in only 32 patients. Salicylic acid levels of >0.01µg/L were detectable in 62.5% of aspirin resistant patients and 70.8% in responders.

## Conclusions

Aspirin resistance among the test cohort is 24.4%. These patients are at greater risk of developing recurrent vascular events in spite of being on aspirin and may benefit by a dose increment. We suggest further studies with larger numbers of patients.

